# Influence of infertility-associated autoantibodies and sexually transmitted infections on implantation success rates after ICSI

**DOI:** 10.5935/1518-0557.20250157

**Published:** 2025

**Authors:** Natalia Bergamo Saraiva Bacarov, Tabea Hammes, Jens Miguel Warnecke, Rafaela Hidalgo, Ana Carolina Bernardo, Claudia Fideles, Letícia Dargenio Garcia, Caio Parente Barbosa, Michel Moraes Soane, Denise Maria Christofolini

**Affiliations:** 1 EUROIMMUN Brasil. Cerâmica, São Caetano do Sul, São Paulo, Brazil; 2 Centro Universitário FMABC, Santo André, São Paulo, Brazil; 3 Institute for Experimental Immunology, affiliated with EUROIMMUN Medizinische Labordiagnostika AG, Luebeck, Germany; 4 IDEIA FÉRTIL Institute of Reproductive Health - Center for Studies in Genetics and Human Reproduction. Santo André, São Paulo, Brazil

**Keywords:** infertility, *in-vitro* fertilization, sexually transmitted infections

## Abstract

**Objective:**

Preimplantation genetic testing improves rates of assisted reproductive technologies success by selecting euploid embryos, reducing implantation failures and miscarriages. However, some factors in women may also impact outcomes, though systematic studies on autoantibodies and implantation success are lacking. In this study, we determined the presence of specific autoantibodies related to infertility and specific antibodies to sexually transmitted infections pathogens among women undergoing intracytoplasmic sperm injection including preimplantation genetic testing and investigated their effect on implantation success.

**Methods:**

Plasma samples from 86 women undergoing in vitro fertilization procedures by intracytoplasmic sperm injection and preimplantation genetic testing were tested for the presence of specific autoantibodies against the ovaries, placenta, uterus, and sperm as well as specific antibodies against *Mycoplasma hominis, Ureaplasma urealyticum, Treponema pallidum, Chlamydia Trachomatis,* Herpes simplex virus 1 and Herpes simplex virus 2. The prevalences of these antibodies were compared between women with successful versus unsuccessful implantation after embryo transfer.

**Results:**

The prevalence of anti-*Ureaplasma urealyticum* antibodies was significantly higher (Fisher’s exact test, *p*=0.028) in women with unsuccessful implantation after intracytoplasmic sperm injection procedures (62.0%), compared to women with successful implantation (36.1%).

**Conclusions:**

The results of this study indicate that a past infection with *Ureaplasma urealyticum* might lead to an increased risk for implantation failure after intracytoplasmic sperm injection procedures. Thus, the detection of IgG antibodies against *Ureaplasma urealyticum* could potentially be used in the future to identify women with an increased risk for implantation failure. However, our findings need to be confirmed in larger cohorts.

## INTRODUCTION

Infertility is a disease in which the effected patient is not able to establish a clinical pregnancy after a duration of 12 months of unprotected, regular sexual intercourse or if the patient’s capacity to reproduce is impaired (Zegers-Hochschild *et al.*, 2017). Of couples within their reproductive ages, infertility is estimated to affect approximately 8 - 12% of couples worldwide (Vander Borght & Wyns, 2018). A broad variety of factors causing infertility has been described in the literature, including maternal or paternal age, endocrine dysfunctions, autoimmune diseases, anatomical abnormalities, genetic abnormalities, and infections affecting the male and female reproductive system (Yatsenko & Rajkovic, 2019). Approximately 30% of infertile couples are diagnosed with unexplained infertility, a condition in which no abnormalities in the female or male reproductive systems are identified (Guideline Group on Unexplained Infertility *et al.*, 2023).

Assisted reproductive technologies (ART), such as in-vitro fertilization (IVF) or intracytoplasmic sperm injection (ICSI), are used to treat infertility. Implantation of a fertilized egg into the uterus is a critical step during ART. The implantation success depends on multiple factors, for example on a competent blastocyst, receptive endometrium and successful crosstalk between the embryonic and maternal interfaces (Franasiak *et al.*, 2021). When performing ICSI, in which a single sperm is directly injected into the unfertilized egg cell, preimplantation genetic testing (PGT) can be performed prior to embryo transfer to gain information on the genetic health of the embryos and subsequently select the most promising embryo for the transfer (Franasiak & Scott, 2017). Different PGT approaches are used in clinical practice, for example PGT for aneuploidy (PGT-A) or PGT for chromosomal structural rearrangements (PGT-SR) (ESHRE PGT Consortium Steering Committee *et al.*, 2020). It has been shown that PGT can increase the success rate of ART by reducing the rates of implantation failure, miscarriage and congenital anomalies, especially in populations with advanced maternal age, where the aneuploidy rate is higher, or in cases of recurrent pregnancy loss (Varga *et al.*, 2019; Greco *et al.*, 2020; Morales, 2024). Despite the benefits of PGT, there is still controversy regarding the effectiveness of PGT in improving pregnancy success and live birth rates in all populations, especially in younger women and those with normal fertility (Morales, 2024).

Franasiak & Scott (2017) concluded that selection of an euploid blastocyst for implantation does not always lead to successful implantation. Several factors other than genetic abnormalities of the fetus may be associated with implantation failure, including maternal immunological and infectious factors (Franasiak & Scott, 2017).

The knowledge on mechanisms behind the development of autoimmune diseases is limited, but exposure to external factors (such as infections, environmental and hormonal factors) and the response of the immune system to these factors can influence the susceptibility of an individual to autoimmune diseases (Ngo *et al.*, 2014). Some of the more common autoimmune diseases like Systemic Lupus Erythematosus (SLE), Type- 1-Diabetes or Hashimoto’s Thyroiditis are well-known causes of impaired fertility (Quintino-Moro *et al.*, 2014; Lin *et al.*, 2018; Stamm *et al.*, 2022). In addition, a link between the presence of autoantibodies without manifestation of the associated autoimmune disease and infertility has been suggested previously (Carp *et al.*, 2012). In this context, there is evidence that antibodies directed against various reproductive tissues and cells may also cause infertility. As an example, Kirshenbaum & Orvieto (2019) found that anti-ovarian antibodies are common amongst women suffering from premature ovarian insufficiency (POI). Also, there is an ongoing controversy, whether anti-sperm antibodies (ASA) play a role in infertility by impairing sperm motility, especially since these have been reported to be significantly increased in both infertile men and women (Nagaria *et al.*, 2011; Gupta *et al.*, 2022).

In addition to the influence of autoimmunity-related processes on fertility, Reese (2024) highlighted the importance of sexually transmitted infections (STIs) in reproductive health. Most active and past STIs can be diagnosed using a combination of tests for direct pathogen detection, like reverse transcription-polymerase chain reaction (RT-PCR) or serological tests; for example testing for pathogen-specific antibodies (Caruso *et al.*, 2021). A considerable number of STIs, including those caused by *Chlamydia trachomatis* or *Neisseria gonorrhoeae*, frequently manifest without symptoms in women, which can lead to a delay in or missed diagnosis and onset of treatment (Van Gerwen *et al.*, 2022). If left untreated, STIs can spread to the upper reproductive organs like the uterus, fallopian tubes or ovaries, leading to pelvic inflammatory disease (PID), which can ultimately result in infertility due to damage or scarring of the affected organs and tissues (Tsevat *et al.*, 2017). In addition, it has been described that STIs can have a negative impact on IVF outcomes, with a higher rate of observed complications like implantation failures or miscarriages (Muller *et al.*, 2015; Zhang *et al.*, 2022).

Despite the continuously increasing know-how in the field of reproductive medicine, knowledge on the influence of the immune system on fertility remains incomplete, especially in the context of autoimmune processes involving reproductive organs. To improve fertility diagnostics and treatment success in the future, it is essential to gain further insights into the pathophysiology behind these mechanisms.

This study aimed to analyze the presence of anti-ovary, anti-placenta, anti-uterus, and anti-sperm antibodies as well as of antibodies directed against STI pathogens among women undergoing euploid embryo transfer after ICSI. Immunofluorescence tests (IIFT) and enzyme-linked immunosorbent assays (ELISA) were used to detect these antibodies, and the results were compared between women with successful versus unsuccessful implantation.

## MATERIAL AND METHODS

We ran a cross-sectional study in which plasma samples were collected from 86 women undergoing fertility treatment at the Ideia Fértil Institute of Reproductive Health - Center for Studies in Genetics and Human Reproduction of ABC (Santo André, São Paulo, Brazil). The study was approved by the local Research Ethics Committee of the medical school of FMABC, São Paulo, Brazil (number 5.139.008).

**Inclusion criteria:** All participants were females, not older than 40 years of age and underwent ICSI and PGT-A or PGT-SR for euploid embryo selection at Ideia Fértil Institute. Only women who transferred embryos from fertilizations carried out with fresh sperm samples obtained by ejaculation were enrolled. Women gave their written informed consent.

**Exclusion criteria:** Women with signs of cervicitis at the time of sample collection, deep endometriosis, or persistent uterine pathogenesis were excluded. Women were excluded if the partner had severe male infertility. Women who chose to transfer more than one embryo, aneuploid embryos, or those with chromosomal mosaicism were excluded.

As there were no data investigating the prevalence of the used antibodies in the Brazilian population, we performed a pilot test in our sample considering ASA (anti sperm antibody). Then, a sample size calculation was performed with the assumption of a prevalence of ASA of 9,3% (standard error of 0,031) among infertile women. Selecting a confidence interval of 95%, a minimum of 80 participants was required to obtain statistically significant results (Miot, 2011).

Samples were collected only during cycles in which euploid blastocysts would be transferred. Blood collection was performed on the third day after the start of the luteal phase, which was supported by progesterone injection according to the treatment protocol. Blood samples were collected in a collection tube with EDTA (Sarstedt brand, model S-Monovette 2.6mL EDTA KE) at Ideia Fértil Institute. After collection, the samples were transferred and stored at -20°C until analysis.

The women were retrospectively divided into two groups depending on the outcome of implantation. Women with either a positive β-human chorionic gonadotropin (β-hCG) test or an ultrasound scan showing a live fetus four to six weeks after embryo transfer confirming successful implantation were assorted to the “successful implantation” group. Participants in the “unsuccessful implantation” group had a negative result on the β-hCG test or ultrasound without embryo and gestational sac.

Presence of IgG autoantibodies directed against tissue of the ovaries, placenta and uterus, as well as ASA was investigated by using the Infertility Mosaic 7 IVD kit (EUROIMMUN Medizinische Labordiagnostika AG, Germany). The plasma samples were processed and analyzed manually, according to manufacturer’s instructions for use.

Additionally, the presence of IgG antibodies against the following pathogens causing sexually transmitted infections (STI antibodies) was evaluated by ELISA commercial kit as described: *Mycoplasma hominis* and *Ureaplasma urealyticum* (Mycoplasma hominis, Ureaplasma urealyticum mosaic KIT, EUROIMMUN);*Treponema pallidum* [Treponema pallidum IgG (FTA-ABS) KIT, EUROIMMUN]; Herpes simplex virus 1 (HSV 1) and Herpes simplex virus 2 ( HSV-2) [Anti-HSV-1 (gC1) ELISA IgG and Anti-HSV-2 (gC2) ELISA IgG KIT, EUROIMMUN]; and *Chlamydia trachomatis* (Anti-Chlamydia trachomatis ELISA Advanced IgG KIT, EUROIMMUN).

### Statistical analysis

For a descriptive data analysis, qualitative variables were presented by absolute and relative frequencies. To compare the qualitative variables, Fisher’s Exact test was used, and a confidence interval of 95% was adopted. The results are shown in Tables 1 and 2.

**Table 1:** Comparison of implantation success after embryo transfer and the number of positive and negative tests for antibodies against reproductive organs and spermatozoa.

Antibody	Unsuccessful implantation		Successful implantation		p-value (Fisher’s exact test)
	Antibody Positive	Antibody Negative	Antibody Positive	Antibody Negative	
Anti-ovarian	1	49	0	36	1.000
Anti-sperm	6	44	2	34	0.459
Anti-uterus	0	50	0	36	1.000
Anti-placenta	0	50	0	36	1.000

**Table 2:** Comparison of implantation success after embryo transfer and the number of positive and negative tests for IgG STI antibodies.

Antibody and method test	Unsuccessful implantation		Successful implantation		p-value (Fisher’s exact)
	Antibody Positive	Antibody Negative	Antibody Positive	Antibody Negative	
Anti- *Treponema pallidum* (IIFT)	6	44	2	34	0.459
Anti- *Mycoplasma hominis* (IIFT)	24	26	14	22	0.510
Anti- *Ureaplasma urealyticum* (IIFT)	31	19	13	23	0.028
Anti- *Chlamydia trachomatis* (ELISA)^1^	0	47	3	28	0.059
HSV-1 (ELISA)^1,2^	41	4	27	2	1.000
HSV-2 (ELISA)^1,3^	9	36	10	20	0.279

1 8 samples did not have sufficient volume to perform this test and were excluded from this analysis.

2 4 borderline results were excluded.

3 3 borderline results were excluded.

The statistical analyses were performed using the Stata statistical program, version

14.0. (Stata, 2015). To demonstrate a statistically significant difference between the analyzed variables, the *p*-value had to be <0.05 in the Fisher’s Exact test. Borderline results were excluded for Fisher’s Exact test.

## RESULTS

The women participating in the study were aged between 31 and 40 years old, with a median of 37 years old. All participants underwent ICSI, with exclusive transfer of euploid blastocysts. Of the 86 women, 65 had primary infertility, 14 had secondary infertility, four had had endometritis previously, two had uterine polyps surgically removed, and one had a monogenic disease.

36 of the 86 women (41.9%) had a successful implantation after embryo transfer and were assigned to the “successful implantation” group. The remaining 50 women (58.1%) did not have a successful implantation and were assigned to the “unsuccessful implantation” group. The median age of women in the “successful implantation” group was 36 years old (95% CI: 34-38), compared to 37 years old (95% CI: 36-39) in the “unsuccessful implantation” group. There was a statistically significant difference in the age of women between the two groups (independent t-test, *p*<0.05).

Considering the entire sample, we observed the frequency of 9.30% positive for ASA and 1.16% were positive for anti-ovarian antibodies. No anti-uterusor anti-placentaantibodies were detected. There was no statistically significant difference in the prevalence of ASA and anti-ovarian antibodies between women with successful versus unsuccessful implantation after embryo transfer (Fisher’s Exact test, Table 1).

Eight out of the 86 women were positive for anti-*Treponema pallidum* antibodies, 38 for anti-*Mycoplasma hominis*-antibodies; 43 for anti-Ureaplasma urealyticum antibodies; three for anti-*Chlamydia trachomatis* antibodies; 68 for anti-HSV-1 antibodies, and 19 for anti-HSV-2 antibodies. According to the results of the Fisher’s Exact test, there was a statistically significant (*p*=0.028) higher prevalence of anti-*Ureaplasma urealyticum* antibodies in the unsuccessful implantation group (62.0%), compared to the successful implantation group (36.1%) (Table 2). No statistically significant difference between the other STI antibodies was found in women with successful versus unsuccessful implantation (Table 2).

## DISCUSSION

Treatment options and diagnostic methods for infertility have been continuously improving over the last decades, but still, in approximately 30% of patients with infertility, the underlying cause for the disease remains unclear, and implantation failure or miscarriages remain a challenge when using ART (Ma *et al.*, 2022; Graham *et al.*, 2023). In this study we aimed to analyze the effect of the presence of autoantibodies directed against reproductive tissues, ASA and STI antibodies on implantation rates after ICSI and PGT in 86 women, to gain further insights into the effect of the immune system on ART success.

Concerning autoantibodies directed against reproductive tissues and ASA, we found a prevalence of 9.30% for ASA and 1.16% for anti-ovarian antibodies. No antiuterus or anti-placenta antibodies were detected. The detected ASA prevalence is in line with previous published prevalences of ASA among infertile women, although some publications report a much higher prevalence of around 40% (Nagaria *et al.*, 2011; Yasin *et al.*, 2016; Ata *et al.*, 2021). The prevalence of anti-ovarian antibodies was lower than what has previously been published; with prevalences of about 30% in infertile women (Luborsky *et al.*, 1999). The difference in observed prevalences might be due to non-standardized testing for autoantibodies directed against components of the reproductive system. Thus, the obtained results and reported prevalences largely depend on the method used for the analysis.

There was no statistically significant difference in the ASA or anti-ovarian antibody prevalence between women with successful versus unsuccessful implantation after euploid embryo transfer; which implies that the presence of these antibodies does not increase the risk for implantation failure when transferring euploid embryos after ICSI. This is in line with previous findings, that as of today, there is no clear evidence that autoantibodies are directly causing implantation failure, except for a strong correlation of the presence of antiphospholipid antibodies and implantation failure (Bashiri *et al.*, 2018). For ASA, this is also in line with the recommendations published in the most recent guideline on unexplained infertility by the European Society of Human Reproduction and Embryology (Guideline Group on Unexplained Infertility *et al.*, 2023). In this guideline, ASA testing in serum is not recommended in the diagnostic workup of infertility in women although the prevalence of ASA seems to be higher in infertile women compared to the general population, due to a lack of evidence that the test result would influence the decision on an outcome of an applied personalized treatment regimen. A similar statement can be found regarding anti-ovarian antibody testing in the new ESHRE guideline for the management of POI, in this guideline it is noted that the prevalence of anti-ovarian antibodies seems to be higher in patients with POI, but due to a lack in evidence regarding the clinical relevance and impact on treatment decisions, routine testing for these antibodies is not recommended (Luborsky *et al.*, 2000; Panay *et al.*, 2024). Thus, further research on the impact of autoantibodies directed against reproductive tissues and ASA on female fertility is needed to assess, whether testing for these antibodies in the workup of infertility would be beneficial to ultimately improve personalized ART treatment regimens.

Regarding the analysis of STI antibodies amongst all women included in the study, the most prevalent ones were antibodies against HSV-1 with a prevalence of 91.89%, followed by antibodies against *Ureaplasma urealyticum* (51.16%), *Mycoplasma hominis* (44.19%), HSV-2 (25.33%), *Treponema pallidum* (9.30%) and *Chlamydia trachomatis* (3.85%). The prevalences of antibodies against HSV-1, HSV-2 and *Treponema pallidum* were in the range of what has been observed in general population studies in Brazil (Clemens & Farhat, 2010; Warnecke *et al.*, 2020). The detected prevalence of antibodies against *Chlamydia trachomatis* was lower compared to previously published data for the general population and for women with infertility (Claman *et al.*, 1997; Warnecke *et al.*, 2020). This might be the result of the ELISA test that was used for the analysis, which has been improved to reduce cross-reactivity of antibodies against other Chlamydia subspecies, especially Chlamydia pneumoniae which occurs in much higher incidence rates compared to Chlamydia trachomatis, thus reducing the number of false positive results. The relatively high prevalences for antibodies against *Ureaplasma urealyticum* and *Mycoplasma hominis* is in line with previous publications on the prevalence of active infections with these pathogens Rodrigues *et al.* (2011). In this context, it has been observed that when analyzing antibodies against *Ureaplasma* and *Mycoplasma* subspecies, cross-reactivity is a possibility that must be considered when interpreting the results. We did, however, observe a significantly higher prevalence of antibodies against *Ureaplasma urealyticum* in the unsuccessful implantation group (62.0%) compared to the successful implantation group (36.1%). In a meta-analysis, Tantengco *et al.* (2021) observed that female infertility was significantly associated with genital infections with *Mycoplasma genitalium, Mycoplasma hominis,* and *Ureaplasma urealyticum*. The same study demonstrated that inflammation caused by these pathogens could result in scarring, cell death, and tubal occlusion - which can be a potential explanation of how these organisms may contribute to female infertility. Our results suggest that a past infection with *Ureaplasma urealyticum* might not only increase the risk for infertility, but also increase the risk for implantation failure when transferring euploid embryos in ICSI procedures. Previous studies have shown that an infection with *Ureaplasma urealyticum* can cause chronic endometritis, a chronic lowgrade inflammation of the endometrium, that seems to be associated with recurrent implantation failure (Johnston-MacAnanny *et al.*, 2010; Franasiak *et al.*, 2018). This might be a possible explanation for our findings and supports the findings of previous research recommending that infections should be considered in the diagnostic workup of recurrent implantation failure (Ma *et al.*, 2022; ESHRE Working Group on Recurrent Implantation Failure *et al.*, 2023). We did not find evidence that a past infection with any of the other investigated STIs has an impact on implantation rates, although this has been suggested by others (Wang *et al.*, 2015; Zhang *et al.*, 2022). This might be due to the relatively small cohort investigated in our study. More studies are needed to confirm these hypotheses and for determining the mechanisms behind the influence of previous infections with STIs on implantation rates in ART.

The evidence of this study is limited by the number of women included in this study. Thus, our results needed to be confirmed in larger cohorts. Also, women in the “unsuccessful implantation” group were significantly older than women in the “successful implantation” group and maternal age has been shown to impact implantation rates (Ma *et al.*, 2022). Therefore, our findings need to be confirmed in studies investigating cohorts of women that are of the same age or that even age-matched patients. Future studies on the influence of the immune system on ART outcome should also investigate different ART techniques and use additional outcome variables, such as number of oocytes retrieved after ovarian stimulation, occurrence of recurrent implantation failure or cumulative live birth to be able to determine which step within a certain ART procedure might be affected by which immunological feature.

## CONCLUSION

Successful implantation after embryo transfer is one of the most critical steps in ART. Even when transferring euploid embryos, implantation failure remains to be a common complication. Present evidence suggests that a previous infection with *Ureaplasma urealyticum* might lead to an increased risk for implantation failure in ART procedures. Thus, the detection of IgG antibodies against *Ureaplasma urealyticum* could potentially be used in the future to identify women with an increased risk for implantation failure, however, targeted treatment options would be required to improve ART success based on the respective test result.
